# Case Report: Chorea-Acanthocytosis Presents as Epilepsy in a Consanguineous Family With a Nonsense Mutation of in VPS13A

**DOI:** 10.3389/fnins.2021.604715

**Published:** 2021-02-10

**Authors:** Fang-Mei Luo, Ming-Xing Deng, Rong Yu, Lv Liu, Liang-Liang Fan

**Affiliations:** ^1^Department of Respiratory Medicine, Diagnosis and Treatment Center of Respiratory Disease, The Second XiangYa Hospital of Central South University, Changsha, China; ^2^Department of Cell Biology, The School of Life Sciences, Central South University, Changsha, China; ^3^Department of Dermatology, Loudi Central Hospital, Loudi, China; ^4^Department of Anesthesiology, The Second Xiangya Hospital, Central South University, Changsha, China; ^5^Hunan Key Laboratory of Animal Models for Human Diseases, Changsha, China

**Keywords:** chorea-acanthocytosis, epilepsy, *VPS13A*, homozygous variant, CHAC

## Abstract

Chorea-Acanthocytosis (ChAc), a rare autosomal recessive inherited neurological disorder, originated from variants in *Vacuolar Protein Sorting 13 homolog A* (*VPS13A*) gene. The main symptoms of ChAc contain hyperkinetic movements, seizures, cognitive impairment, neuropsychiatric symptoms, elevated serum biochemical indicators, and acanthocytes detection in peripheral blood smear. Recently, researchers found that epilepsy may be a presenting and prominent symptom of ChAc. Here, we enrolled a consanguineous family with epilepsy and non-coordinated movement. Whole exome sequencing was employed to explore the genetic lesion of the family. After data filtering, co-separation analysis was performed by Sanger sequencing and bioinformatics analysis, the homozygous nonsense variant (NM_033305.2: c.8282C>G, p.S2761X) of *VPS13A* were identified which could be genetic factor of the patient. No other meaningful mutations were detected. This mutation (p.S2761X) led to a truncated protein in exon 60 of the *VPS13A* gene, was simultaneously absent in our 200 local control participants. The homozygous mutation (NM_033305.2: c.8282C>G, p.S2761X) of *VPS13A* may be the first time be identified in ChAc patient with epilepsy. Our study assisted to the diagnosis of ChAc in this patient and contributed to the genetic diagnosis and counseling of families with ChAc presented as epilepsy. Moreover, we further indicated that epilepsy was a crucial phenotype in ChAc patients caused by *VPS13A* mutations.

## Introduction

Chorea-acanthocytosis (ChAc, OMIM #200150) is a primary neurological disorder characterized by repetitive movements of various parts of the body and abnormal red blood cell shape (Velayos Baeza et al., 1993; Liu et al., 2018; Roulis et al., 2018). As fewer than 1000 cases have been reported worldwide, researchers commonly consider ChAc to be a rare genetic disease (Peikert et al., 2018). Previous studies have revealed that autosomal-recessive mutations in the *Vacuolar Protein Sorting 13 homolog A* (*VPS13A*) gene may be the genetic lesion of ChAc (Velayos Baeza et al., 1993). Epilepsy is also a central nervous system disorder that is described as “a common brain condition that causes repeated seizures.” In addition to recurrent seizures, other symptoms of epilepsy include temporary confusion, staring spells, uncontrollable jerking movements of the arms and legs, and loss of consciousness or awareness (Yuen et al., 2018; Thijs et al., 2019). A recent study revealed that the prevalence of epilepsy was 3.5–6.5 per 1,000 children and 10.8 per 1,000 elderly individuals worldwide (Abramovici and Bagic, 2016). In a recent summary of 5 years of research in patients with VPS13A mutations, ~45% of patients have a history of epileptic seizures, which is consistent with previous studies in ChAc claiming that ~42% of ChAc patients have at least one seizure at some point in their clinical course (Benninger et al., 2016).

In the past decade, the rapidly growing number of genes underlying genetic epilepsy has been attributed to the application of high-throughput sequencing technology. According to the EpilepsyGene database (http://www.wzgenomics.cn/EpilepsyGene/index.php), ~500 genes may be associated with epilepsy, and ~2% of idiopathic epilepsy patients are thought to be monogenic (Ran et al., 2015). Mutations in *Sodium Voltage-Gated Channel Alpha Subunit 1* (*SCN1A*), *Potassium Voltage-Gated Channel Subfamily Q Member 2* (*KCNQ2*), *Cyclin Dependent Kinase Like 5* (*CDKL5*), *Protocadherin 19* (*PCDH19*), and *Proline Rich Transmembrane Protein 2* (*PRRT2*) were the most common genetic lesions of epilepsy (Wang J. et al., 2017; Kang et al., 2019). In addition, recent studies revealed that epilepsy may be a presenting and prominent symptom of ChAc caused by *VPS13A* mutations (Benninger et al., 2016; Weber et al., 2018).

Here, a patient with simultaneous epilepsy and non-coordinated movement was enrolled. Family history indicated that the parents were in a consanguineous marriage. Whole-exome sequencing and Sanger sequencing were applied to explore the candidate genes of the family.

## Case Presentation

**Figure 1:**
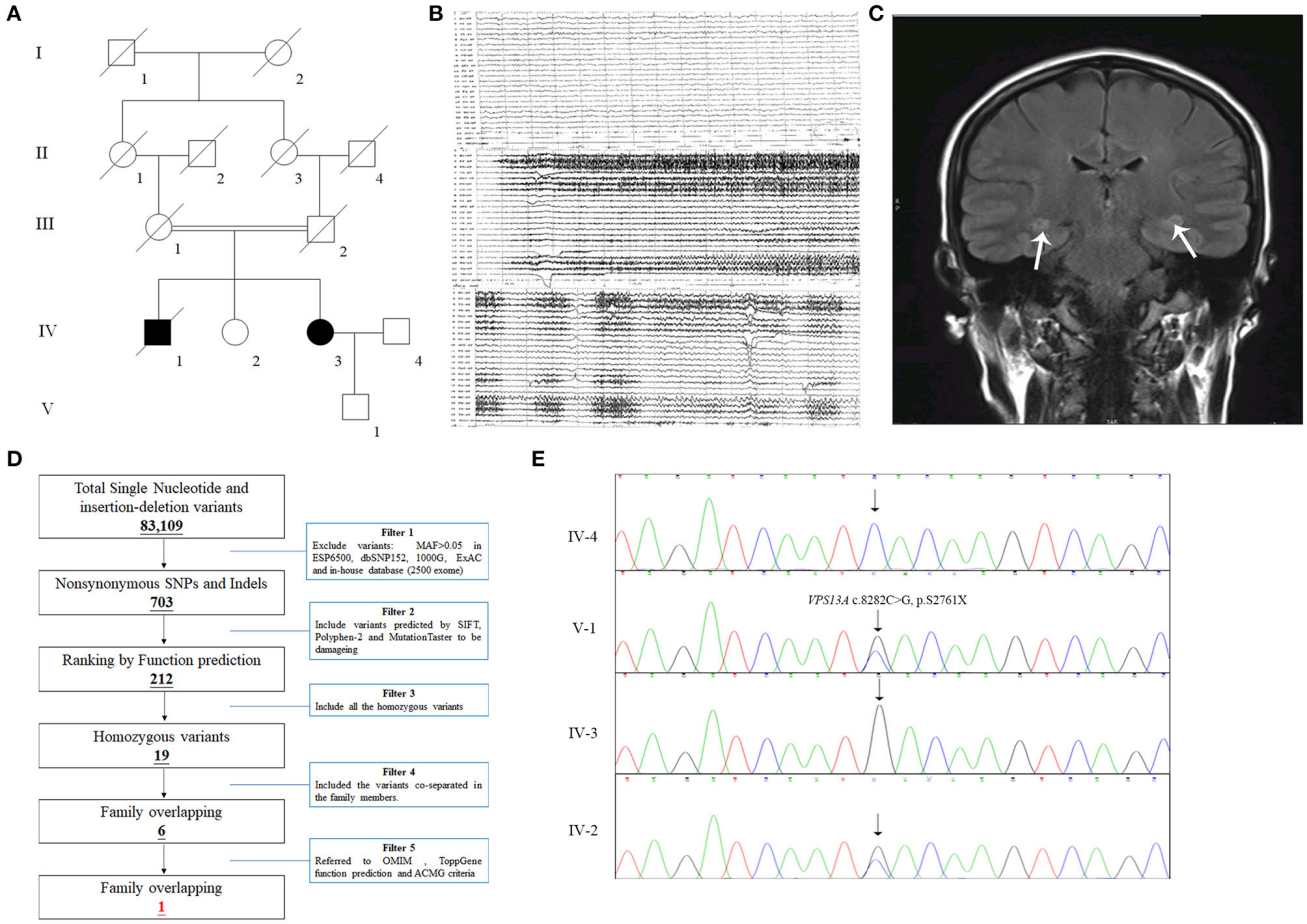
The clinical and genetic data of the family. **(A)** Pedigree of the family with epilepsy. Double lines indicate consanguineous unions. Square denotes male family member, circle represents female family member, slashed symbol indicates deceased family member, fully shaded symbol shows patient with epilepsy, and open symbol presents no symptoms member. **(B)** EEG testing of the IV-3 showed frequent abnormal epileptiform discharges in the right temple, and a small amount of epileptiform discharges in the left; **(C)** MRI scanning of the IV-3 revealed that T2 and T2 flair signals of bilateral hippocampus were slightly higher (the arrows), the hippocampal neuron may be damaged. **(D)** Schematic representation of the filter strategies employed in this study. **(E)** Sanger sequencing validated that the patient IV-3 carried a homozygous mutation (NM_033305.2: c.8282C>G, p.S2761X) of *VPS13A* and her son (V-1) and sister (IV-2) carried the heterozygous mutation.

All 13 family members were investigated in this study (Figure 1A). The peripheral blood samples of one patient (IV-3) and three unaffected family members (IV-2, IV-4, and V-1) were collected. Clinical data, such as magnetic resonance imaging (MRI) and electroencephalogram (EEG), were recorded carefully. In addition, 200 unrelated, ethnically matched healthy controls were used as internal controls to exclude SNPs in local individuals. These healthy controls (male/female: 100/100, age 36.7 ± 8.6 years) lacked ChAc diagnostic features. Each participant underwent thorough examination for clinical diagnosis or exclusion, including general examination, such as EEG and other behavioral testing.

The patient, a 43-year-old female, was admitted to the hospital due to lung infection. However, the patient presented recurrent seizures during hospitalization. EEG testing showed frequent abnormal epileptiform discharges in the right temple and a small amount of epileptiform discharges in the left temple (Figure 1B). MRI scanning revealed that T2 and T2 flair signals of the bilateral hippocampus were slightly higher, and hippocampal neurons may be damaged (Figure 1C). Serum creatine kinase, l-lactate dehydrogenase and alpha hydroxybutyrate dehydrogenase levels were all within normal ranges (Table 1). Medical history revealed that the patient presented mild non-coordinated mandibular movement and upper limb movements for seven months. A family history survey indicated that her mother and father were intermarried, and her brother (IV-1) also presented non-coordinated mandibular movement and upper limb movements at 37 years old according to the family members' descriptions. No other family members presented similar symptoms.

**Table 1:** The clinical summary of the patient.

**Items**	**IV-3**
Age (year)	43
Family history	Intermarriage
Primary symptoms	Mild mandibular movement and upper limb movements
Dystonia distribution	Upper limbs
Seizure	+++
Psychiatric symptoms	Apathy
CK (38-170U/L)	152
LDH (109-245U/L)	174
HBDH (72-182U/L)	137
ALT (5-40U/L)	17
AST (8-40U/L)	16
Acanthocyte ratio of CBC	18%
EEG testing	Frequent abnormal epileptiform discharges in the right temple, and a small amount of epileptiform discharges in the left
MRI	T2 and T2 flair signals of bilateral hippocampus were slightly higher, the hippocampal neuron may be damaged

*CK, creatine kinase; LDH, l-lactate dehydrogenase; HBDH, alpha hydroxybutyrate dehydrogenase; ALT, alanine transaminase/glutamic-pyruvic transaminase; AST, aspartate transaminase/glutamic–oxaloacetic transaminase; CBC, complete blood count; EEG, electroencephalogram; MRI, Magnetic Resonance Imaging*.

### Genetic Analysis

Genomic DNA was extracted from peripheral blood lymphocytes of four family members with PureLink™ Genomic DNA Mini Kit (Invitrogen, the USA). We selected the proband's DNA to perform the whole exome sequencing. Whole exome sequencing services were provided by the Novogene Bioinformatics Institute (Beijing, China). Exomes were captured by Agilent SureSelect Human All Exon V6 kits, and next-generation sequencing was conducted with an Illumina HiSeq X-10 system as we previous described (Yu et al., 2017; Wang et al., 2020).

The strategies of data filtering are as follows (Figure 1D): (a) Non-synonymous SNPs or frameshift-causing INDELs with an alternative allele frequency > 0.05 in the NHLBI Exome Sequencing Project Exome Variant Server (ESP6500), dbSNP152 (http://www.ncbi.nlm.nih.gov/projects/SNP/index.html), the 1000 Genomes project (http://www.1000genomes.org/), the ExAC database (http://exac.broadinstitute.org) or in-house exome databases of Novogene (2500 exomes) were excluded; (b) the filtered SNVs and INDELs, predicted by SIFT (http://sift.jcvi.org/), Polyphen2 (http://genetics.bwh.harvard.edu/pph2/), and MutationTaster (http://www.mutationtaster.org/) to be damaging, were remained; (c) all the homozygous mutations were remained; (d) Co-segregation analysis was conducted in the family.

Here, 99.3% coverage of the target regions and 97.70 × sequencing depth were achieved for the proband. Approximately 83,109 variants were found in the proband. Via the abovementioned filtering method, six homozygous mutations were detected (Table 2). Further Sanger sequencing and bioinformatics analysis indicated that only the nonsense mutation (NM_033305.2: c.8282C>G, p.S2761X) in *the VPS13A* gene may be the genetic lesion of the patient (Figure 1E). No other potential pathogenic mutations for epilepsy diseases were found. The novel mutation, generating a truncated protein in exon 60 of the *VPS13A* gene, was absent in our 200 local control participants. Bioinformatics programs predicted that this mutation (NM_033305.2: c.8282C>G, p. S2761X) is a pathogenic mutation located in an evolutionarily conserved site of the VPS13A protein. According to ACMG guidelines (Richards et al., 2015), this mutation belongs to pathogenic (PVS1+PM2+PM3).

**Table 2:** The filtered homozygous mutations of whole exome sequencing.

**Gene**	**Transcript variant**	**Protein variant**	**SIFT**	**Polyphen-2**	**Mutationtaster**	**OMIM Clinical Phenotype**	**ToppGene function**	**ACMG statement**
*LMF2*	NM_033200.3: c.1370G>A	p.R457H	D	P	D	-	Digestion of dietary lipid	Uncertain significance (PM2+PP3+BP4)
*NDUFAF7*	NM_144736.5: c.581T>A	p.I194N	D	D	D	AD: pathologic myopia	Peptidyl-arginine modification	Uncertain significance (PM2+PP3+BP5)
*VPS13A*	NM_033305.3: c.8282C>G	p.S2761X	-	-	D	AR: Choreoacanthocytosis	Loss of basal ganglia neurons	Pathogenic (PVS1+PM2+PM3)
*RPUSD1*	NM_058192.3: c.370G>C	p.E124Q	D	P	D	-	Pseudouridine synthesis	Uncertain significance (PM2+PP3+BP4)
*GDPD4*	NM_182833.3: c.625-2A>C	-	-	-	D	-	Phosphoric diester hydrolase activity	Uncertain significance (PM2+PP3+BP4)
*DSP*	NM_004415.4: c.1_2insC	p.M1fs	-	-	D	AD: Cardiomyopathy	Cardiac muscle cell-cardiac muscle cell adhesion	Pathogenic (PVS1+PM2+PM4)

*D, Disease-causing; P, Possible damaging; AD, Autosomal Dominant; AR, Autosomal Recessive; PVS, pathogenic very strong; BP, pathogenic benign; PP, pathogenic supporting; PM, pathogenic moderate; LMF2, Lipase Maturation Factor 2; NDUFAF7, NADH Dehydrogenase (Ubiquinone) Complex I, Assembly Factor 7; VPS13A, Vacuolar Protein Sorting 13 homolog A; RPUSD1, RNA Pseudouridine Synthase Domain Containing 1; GDPD4, Glycerophosphodiester Phosphodiesterase Domain Containing 4; DSP, Desmoplakin*.

### Discussion

After data filtering, six homozygous mutations remained, including the mutation (NM_033305.2: c.8282C>G, p.S2761X) of *VPS13A*. In the other five homozygous mutations, previous studies have revealed that mutations in *NDUFAF7* may lead to pathologic myopia (Wang B. et al., 2017), and mutations in *DSP* may be linked to cardiomyopathy (Singh et al., 2018). Hence, we exclude these two mutations (NM_144736.5: c.581T>A and NM_004415.4: c.1_2insC). The *GDPD4* gene may be related to obesity (Kaewsutthi et al., 2016), and *RPUSD1* may be related to the methylome at birth and adolescence (Alfano et al., 2019). Hence, mutations in both *GDPD4* and *RPUSD1* may have extremely low effects on epilepsy. Few studies on the *LMF2* gene are available, and ToppGene predicted that this gene may be related to digestion of dietary lipids. In our candidate mutations, the VPS13A mutation was a nonsense mutation, which has been reported in ChAc. In recent years, an increasing number of studies have revealed that epilepsy may be a presenting and prominent symptom of ChAc caused by VPS13A mutations (Benninger et al., 2016; Weber et al., 2018). In addition, according to ACMG criteria, only *VPS13A* and *DSP* mutations belong to the pathogenic level. Synthetic judgments indicated that the mutation (NM_033305.3: c.8282C>G) of *VPS13A* was the genetic lesion of the family.

The human *VPS13A* gene encoding a vacuolar protein sorting-associated protein named chorein is located on chromosome 9q21.2 and consists of 72 exons spanning ~244.2 kilobases (kb) (Velayos-Baeza et al., 2004). Chorein controls steps in the cycling of proteins through the trans-Golgi network to endosomes, lysosomes and the plasma membrane (Kumar et al., 2018; Yeshaw et al., 2019). In 2001, Rampoldi et al. identified 16 different mutations of *VPS13A* in 11 unrelated families with ChAc, indicating that *VPS13A* mutations might be involved in the etiology of this autosomal recessive disorder (Rampoldi et al., 2001). Since then, more than 100 *VPS13A* gene mutations have been identified in ChAc patients (Shen et al., 2017). However, in recent years, an increasing number of studies have revealed that ChAc caused by *VPS13A* mutations may present a prominent symptom of epilepsy or even present only an isolated epilepsy phenotype (Al-Asmi et al., 2005; Benninger et al., 2016; Weber et al., 2018). In this study, we identified a homozygous nonsense variant (NM_033305.2: c.8282C>G, p.S2761X) of *VPS13A* in a consanguineous family with epilepsy, which further demonstrated that *VPS13A* mutation may exhibit an epilepsy phenotype.

The human VPS13A protein contains several conserved domains, including the N-terminal chorein domain (aa 3-119), SHR-binding domain and APT1 domain (aa 2522-2856) (Rzepnikowska et al., 2017; Kumar et al., 2018; Soczewka et al., 2019). Previous studies have indicated that phosphatidylinositol 3-phosphate (PI3P) can regulate the functioning of VPS13A, both in protein trafficking and actin cytoskeleton organization (Rzepnikowska et al., 2017). VPS13A can interact with actin and regulate the level of phosphatidylinositol 4-phosphate (PI4P) in the membranes of neuronal cells (Rzepnikowska et al., 2017). In our study, the nonsense mutation (NM_033305.2: c.8282C>G, p. S2761X) may generate a truncated protein without an integrated APT1 domain, which can disrupt the structure and function of the VPS13A protein and affect the level of phosphatidylinositol 4-phosphate (PI4P) in neuronal cell membranes, disrupt the structure and function of neurons, and finally present T2 and T2 flair signals in the bilateral hippocampus that increase on MRI, which indicates damage to hippocampal neurons.

Simultaneously, recent studies have also indicated that the VPS13A protein may also regulate the storage and release of calcium (Yu et al., 2016; Pelzl et al., 2017a,b). VPS13A can interact with and foster stimulation of phosphoinositide-3-kinase (PI3K), which may further activate NF-κB with subsequent upregulation of the Ca^2+^ channel (Sukkar et al., 2018). Studies have indicated that the functional connection between VPS13A and calcium signaling is a possible target for chemical intervention in ChAc (Soczewka et al., 2019). Lithium treatment can reverse the enhanced neuronal apoptosis of ChAc fibroblasts, which is caused by decreased SOCE, a Ca^2+^-channel accomplishing store-operated Ca^2+^ entry (Pelzl et al., 2017b; Sukkar et al., 2018). In our study, the mutation (NM_033305.2: c.8282C>G, p. S2761X) changed the structure of VPS13A and may affect the interaction between VPS13A and PI3K, disrupting the release of calcium signals in neurons and leading to epilepsy and one calcium disorder (Steinlein, 2014), presenting frequent abnormal epileptiform discharges in EEG.

The mutation (NM_033305.2: c.8282C>G, p. S2761X) of *VPS13A* was first identified in a ChAc patient who carried the heterozygous mutation p. S2761X and did not present epilepsy phenotypes (Shen et al., 2017). Here, we may be the first detect the homozygous mutation (NM_033305.2: c.8282C>G, p. S2761X) of *VPS13A* in a Chinese consanguineous family member who presented epilepsy phenotypes. Recently, an increasing number of studies have revealed that epilepsy is a crucial presentation in patients with *VPS13A* mutations (Benninger et al., 2016; Weber et al., 2018). Therefore, our study provided a new epilepsy case caused by a VPS13A mutation, which may further confirm the epilepsy phenotype in ChAc and expand the phenotype of the mutation (NM_033305.2: c.8282C>G, p.S2761X) of *VPS13A*. Some limitations in our study should be noted. For instance, we only enrolled one patient with ChAc who presented with an epilepsy phenotype. The data filtering of whole-exome sequencing was insufficient to exclude unknown mutations. Additional functional analysis of the VPS13A protein with this mutation is recommended and may result in additional information about the pathogenetic mechanism of ChAc presented as epilepsy.

## Conclusions

In summary, a homozygous missense mutation (NM_033305.2: c.8282C>G, p. S2761X) in the *VPS13A* gene was identified in a consanguineous Chinese family with epilepsy. To our knowledge, this is the first report of the homozygous *VPS13A* c.8282C>G mutation in a ChAc patient. Our study further confirmed that epilepsy was an important phenotype in patients with mutated *VPS13A*, which may extend the genotype-phenotype relationship between mutations in the VPS13A gene and clinical findings of ChAc and help us understand how the different mutations could contribute to different symptoms of the same disease. In addition, our study may be helpful in the genetic counseling of patients with *VPS13A* mutations.

## Data Availability Statement

The datasets for this article are not publicly available due to concerns regarding participant/patient anonymity. Requests to access the datasets should be directed to the corresponding author.

## Ethics Statement

The studies involving human participants were reviewed and approved by the Ethics Committee of the Second Xiangya Hospital of Central South University, Changsha, China. The patients/participants provided their written informed consent to participate in this study. Written informed consent was obtained from the individual(s) for the publication of any potentially identifiable images or data included in this article.

## Author Contributions

F-ML and M-XD enrolled the family members. M-XD performed DNA isolation and Sanger sequencing. F-ML and RY performed genetic analysis. F-ML and LL wrote the manuscript. L-LF supported the project. All authors reviewed the manuscript.

## Conflict of Interest

The authors declare that the research was conducted in the absence of any commercial or financial relationships that could be construed as a potential conflict of interest.
