# Corrigendum: Case Report: Chorea-Acanthocytosis Presents as Epilepsy in a Consanguineous Family With a Nonsense Mutation of in VPS13A

**DOI:** 10.3389/fnins.2021.687435

**Published:** 2021-04-30

**Authors:** Fang-Mei Luo, Ming-Xing Deng, Rong Yu, Lv Liu, Liang-Liang Fan

**Affiliations:** ^1^Department of Respiratory Medicine, Diagnosis and Treatment Center of Respiratory Disease, The Second XiangYa Hospital of Central South University, Changsha, China; ^2^Department of Cell Biology, The School of Life Sciences, Central South University, Changsha, China; ^3^Department of Dermatology, Loudi Central Hospital, Loudi, China; ^4^Department of Anesthesiology, The Second Xiangya Hospital, Central South University, Changsha, China; ^5^Hunan Key Laboratory of Animal Models for Human Diseases, Changsha, China

**Keywords:** chorea-acanthocytosis, epilepsy, *VPS13A*, homozygous variant, CHAC

In the published article, there was an error in affiliation 1. Instead of “Department of Respiratory Medicine, Diagnosis and Treatment Center of Respiratory Disease, The Second Xiangtan Hospital of Central South University, Changsha, China,” it should be “Department of Respiratory Medicine, Diagnosis and Treatment Center of Respiratory Disease, The Second XiangYa Hospital of Central South University, Changsha, China.”

The authors apologize for this error and state that this does not change the scientific conclusions of the article in any way. The original article has been updated.

